# On Ether as a Local Application

**Published:** 1866-07

**Authors:** John J. Black

**Affiliations:** Philadelphia


### Selections.

### ON ETHER AS A LOCAL APPLICATION.

By JOHN J. BLACK, M.D., of Philadelphia.

Our attention was drawn to this subject by an article in the Quarterly Summary of the number of this Journal for October 1864, p. 523, on the "Pathology and Treatment of Aphthæ." The author of that article, Dr. Jules Worms, whose paper originally appeared in the *Gazette Hebdomadaire*, concluded from a minute examination of the deposit on the surface of aphthæ, that it consisted of a fatty matter which is not to be found in any other disease of the mouth, and he inferred from the solubility of the exudation in ether, that this article might prove a useful remedy for the affection. Accordingly he resorted to the application, and with the most beneficial results. This remedial agent removes, he states, "the yellowish secretion, a new epithelium promptly forms, and no trace of the superficial ulcers remains beyond slightly increased vascularity of the mucous membranes."

Prompted by this statement, I determined to give the remedy an extended trial, and I shall now endeavor to show, by the results, that ether locally applied is a most efficient remedy not only in aphthous ulcers but also in most of the other diseases of the mucous membrane of the mouth and adjacent parts, in which, according to the researches of Dr. Worms, the deposits are of a non-fatty nature.

*Aphthæ.*—We have used the application in several cases of this disease, and invariably found the affection to yield after a few applications, daily repeated. A camel's-hair brush was dipped in ether an applied freely over the parts; it appeared to smart a little at first, but great relief soon followed. This was certainly marked both in character and point of time, in comparison with that obtained by borax and similar preparations.

"*Thrush.*"—In this disease above all others we have been pleased with the results of the application. The cases presented themselves in the obstetrical wards of the Philadelphia Hospital, Blockley, which of course were fruitful in that disease—containing so many badly nourished children. It was applied directly to the parts, as in aphthæ, with a camel's-hair brush.

At first it produced or seemed to produce a slight difficulty in inspiration, which was soon relieved by a hearty cry of the infant. Of course its presence in the mouth could not have been pleasant, but in no case was it followed by an unpleasant symptom. The deposit was not immediately dissolved, but seemed to disappear gradually, and in most cases after twenty-four hours there was none whatever to be seen, and the one application completed the cure (at least the local cure). In other cases a few spots remained, and if they persisted after another twenty-four hours we repeated the application, and in every instance a cure resulted. These cases were all carefully watched, some of them for several months, and in no case was there the slightest return of the complaint. In from three to four days the mucous membranes became perfectly normal; in the interval from the disappearance of the deposit to this time it presented something of the appearance of erythematous stomatitis without the usual dryness attendant on that affection. Between twenty and thirty cases were treated in this manner, and after the disappearance of the thrush they improved wonderfully. These results tend to strengthen the idea that thrush is a local disease confined to the mouth, or at least that this part only causes inconvenience, and the constitutional troubles as it were radiate from that centre.

"*Ulcero-Membranous Stomatitis.*"—In this disease we have had the opportunity of testing it in three cases. One supervening upon pleurisy died with extensive sloughing of the parts of the jaw involved. Another recovered without any serious trouble, and seemed to have been greatly benefited by the ether. The third case, more serious, also recovered. Here the parts were apparently in a gangrenous condition, and it only differed from true gangrene of the mouth in commencing in, and being more particularly confined to, the gums, without seriously involving the cheek. The sloughs were kept well detached, the parts washed with diluted chlorinated soda, and the ether applied thoroughly morning and evening. A change for the better soon came over the parts, and the patient recovered with the loss of a portion of the alveolar border of the jaw. Of course we combined with this treatment tonics and stimulants to the fullest extent.
to cleanse the teeth, gums, and tongue from sordes, and such accumulations in low fevers, and might possibly produce a permanent change for the better in the secreting apparatus there situated.

There is another trouble in which ether must prove a very valuable remedy, although we have not had the opportunity of testing it. We refer to "herpes præputialis," that annoying and troublesome complaint with which some persons are so much afflicted. It would also possibly prove efficacious in many skin affections, such as eczema, psoriasis, etc., the crusts having first been removed with a poultice.

According to Prof. Wood, ether has been locally applied to neuralgic pains, earache, superficial burns and scalds, and also to aid in the reduction of strangulated hernia; but in all these cases the cuticle was entire. There are doubtless many other conditions in which ether might be beneficially applied as a local agent, and which will doubtless suggest themselves in practice. There is, however, one more condition in which we must refer to its beneficial action, viz., chronic ulcers. We have not had the opportunity recently of observing it in those troublesome lesions, and here beg leave to express our thanks for the following observations made in the surgical wards of the Philadelphia Hospital, Blockley, by our friend Dr. Charles E. Smith, Jr., resident physician in that institution.

"At the suggestion of Dr. J. Black I tried the effects of ether as an application to chronic ulcers in the surgical wards of the Philadelphia Hospital, Blockley. For the first trial I selected ten cases, the ulcers being on the legs, and from one to twelve years' standing; some of them being partially healed during that time, and had reopened. In seven of these ten cases the sores were covered with a dirty yellowish-white exudation, looking as though melted tallow had been poured over them; two were indolent in character with thickened and everted edges; one was in a sloughing condition. The seven first mentioned healed up rapidly in from two to four weeks, leaving a *very* small cicatrix. The indolent ones improved for a time under the use of the ether, but soon ceased to respond, other remedies seeming to finish what it began. The sloughing one remained as it was, the application seeming to have no effect. The patient died of scurvy. After this I tried it upon three more cases in which the tallow exudation was well marked, with the most satisfactory results, they having healed within three weeks. The ether was applied with a camel's-hair pencil, about one drachm painted on every morning like varnish, nothing else

The question here arises whether ether might not act beneficially locally applied to true gangrene?

In acute pharyngitis, the sore throat every day met with, we have found it one of the very best applications, in all its stages. We apply it with a camel's-hair brush; at first it stings for a minute or two, and then a pleasant coolness is experienced in the part, giving most marked relief, and patients almost invariably express themselves as feeling greatly benefited. The most noticeable feature in these cases is, the rapid subsidence of the swelling and tumefaction of the parts, and which the patient never fail to notice. In chronic pharyngitis also it produces the same marked relief, and in specific and non-specific ulcerations of the throat, where the patient is much troubled by the accumulations of mucous and the other secretions, we have found the best plan of treatment to consist in washing out the throat well with a mop dipped in a strong alkaline solution, which dissipates the mucus, etc., and then applying ether to the parts. In chronic laryngitis we have seen benefit derived from inhaling ether, not of course up to the production of anæsthesia. Here an attempt at its local application proves decidedly irritating.

In diphtheria we have not yet had the opportunity of testing the remedy, but this is the disease in which we have always expected to derive the greatest benefit from it. While we do not consider the mere throat manifestations as the sum and substance of diphtheria, nevertheless it is rational to suppose that these exudations, when swallowed, must tend to poison the system anew, and set up exhausting diarrhœas, etc., and looking at them in this light, it is certainly of the greatest importance to get rid of them. It is also, perhaps, of not less importance to get rid of the mechanical impediment which they offer to the breathing, and also of the swelling, which is so often a serious matter. As before stated, we have not had the opportunity of testing it in this disease, but my friend, Dr. D. F. Woods, of this city, has kindly reported to me a case in which he used it, and in which he derived from it all the benefits before mentioned. He said it appeared to disperse the membrane, reduced the swelling, and altogether placed the patient in a more comfortable condition. Dr. Woods also reported to me that he had used it often in the ordinary pharyngitis, tonsillitis, etc., frequently combining nitrate of silver with it, and always with the most marked and decided benefit, surpassing in his estimation all other remedies.

It strikes me at the moment that ether might also be useful being done for them, except supporting the leg with a bandage. They improved from the first application, granulations of a healthy character sprung up almost immediately, and the ulcers healed rapidly, leaving a very slight cicatrix. In one case in particular in which the ulcers had existed for three years, the skin for several inches around them being of a dirty brown color, healed as though by magic, and at the end of a month the coloration had almost entirely disappeared. These experiments have lead me to the conclusion that in chronic ulcers, in which there is an exudation over the surface looking like a false membrane, the ether causes it to disappear and granulatious to spring up, the ulcers healing much sooner than by any other means. In indolent ulcers with raised edges it acts very well for a time as a stimulant, but sooner commences to lose its power. I think the ether acts more decidedly during hot than during cold weather, the ulcers then appearing to respond to it with more certainty."

The ether used in all the foregoing cases was the sulphuric ether, the "Æther," U.S.P.

In regard to the manner in which ether locally applied produces its results, we remark, in the first place, that it is a local stimulus, and appears thoroughly searching and penetrating in its action. Thus then it increases absorption, prevents or dispels congestion, and allows free osmotic action. Again, very probably it changes the nature of the local cell action, which having been turned from its normal channel may thus be driven back to its course. Again, it undoubtedly acts to a great extent chemically. It has been shown by Rokitansky and others that most of these exudative inflammations contain fat, both the mucoid exudative and the fibrinous exudative, the one running into the other, except it be checked. Now the well-known solvent power of ether over fats shows us then that it must act beneficially through its chemical properties. Other chemical changes it doubtless brings about, but of their true nature we are at present unable to determine.

Such then are the results obtained from our short experience in the application of ether as a local remedy, that it is available for much more extended usefulness, we have little doubt; as such then when we commend it to the profession, with the full assurance that it will prove a valuable accession to the aramentarium of the physician.

N.B.—It is well to bear in mind when using ether in any manner that great caution must always be observed to prevent the near approach of flame or heat to it, for by neglecting this point serious accidents may arise.

